# Ultrasound screening for cystic echinococcosis of school-age children in endemic areas of Chile: A pilot cross-sectional study towards integration in the Regional Program for the Elimination of Cystic Echinococcosis 2020–2029

**DOI:** 10.1371/journal.pntd.0013301

**Published:** 2025-07-21

**Authors:** Cristian A. Alvarez Rojas, Tommaso Manciulli, Francesca Tamarozzi, Michele Spinicci

**Affiliations:** 1 Escuela de Medicina Veterinaria, Facultad de Agronomía y Sistemas Naturales, Facultad de Ciencias Biológicas y Facultad de Medicina, Pontificia Universidad Católica de Chile, Santiago de Chile, Chile; 2 Department of Experimental and Clinical Medicine, Università degli Studi di Firenze, Firenze, Italy; 3 Department of Infectious-Tropical Diseases and Microbiology, WHO Collaborating Centre for Strongyloidiasis and other Neglected Tropical Diseases, IRCCS Sacro Cuore Don Calabria Hospital, Negrar di Valpolicella, Verona, Italy; Bayer AG, SWITZERLAND

## Abstract

**Background:**

Cystic echinococcosis (CE) is a public health problem in livestock-breeding areas, including Chile, which adhered to the Pan American Health Organization Regional Program for the Elimination of CE 2020–2029. Abdominal ultrasound (US) screening of school-aged children (SAC) in high-risk areas is envisaged by the Action Plan for Control, Monitoring and Prevention of CE of the Ministry of Health (MoH) of Chile. We implemented pilot US screening and estimated CE prevalence in SAC in three municipalities prioritized by the MoH, to inform about the feasibility of targeting this age group within the activities of the Action Plan.

**Methodology/Principal findings:**

A cross-sectional US screening was carried out in SAC (6–14 years) in Río Hurtado (Coquimbo region), Paillaco (Los Ríos), and Chile Chico (Aysén) municipalities. CE was diagnosed according to the WHO-IWGE recommendations. A total of 873 SAC were screened over 10 working days, with excellent participation (81–93%). Three children had hepatic CE cysts (3/873; 0.3%); the highest prevalence was found in Chile Chico (0.6%, 95% CI 0.2%-2.2%).

**Conclusions/Significance:**

US screening in SAC is technically feasible in Chile. While prevalence of CE in SAC might be too low to allow school-based monitoring and evaluation of a structured control program, US screening in SAC could allow early case-finding and support the implementation of control measures around new diagnoses.

## Author summary

Cystic echinococcosis (CE) is a zoonosis transmitted between dogs and ungulates; in humans, CE is a public health problem where livestock breeding is practiced, including Chile, which adhered to the Pan American Health Organization Regional Program for the Elimination of Cystic Echinococcosis 2020–2029. Abdominal ultrasound (US) screening of school-aged children (SAC) in high-risk areas is envisaged by the Action Plan for Control, Monitoring and Prevention of CE of the Ministry of Health (MoH) of Chile. US screening in SAC is carried out in the Control Program of CE in Río Negro provice, Argentina; furthermore, targeting SAC in control programs for other neglected tropical diseases is common, due to the possibility for an early treatment and the easy accessibility of SAC. We implemented an US screening and estimated CE prevalence in SAC in three municipalities identified by the MoH of Chile as at high risk. Our results showed that US screening in SAC is feasible in Chile; however, prevalence of CE in this age group might be too low to allow school-based monitoring and evaluation of a control program. On the contrary, US screening in SAC could allow early case-finding and support the implementation of control measures around new diagnoses.

## Introduction

Cystic echinococcosis (CE) is a neglected zoonotic disease caused by infection with the larval stage of the parasite *Echinococcus granulosus sensu lato*. Its life cycle develops between canids (most commonly dogs herding livestock or free-roaming dogs) and livestock (most commonly sheep and goats) [1]. Canids, the definitive hosts, develop intestinal infection with the adult parasites, which are small cestodes (2–7 mm) shedding proglottids and eggs through the feces of infected animals. Livestock, intermediate hosts, become infected by ingestion of viable parasite eggs contaminating the environment, and develop the larval stage (CE cyst) in internal organs. When dogs feed on infected livestock offal and ingest fertile larval cysts, the parasite’s life cycle completes. Humans are accidental, dead-end hosts, becoming infected through ingestion of viable parasite eggs. In humans, CE cysts develop mainly in the liver and lungs, although all organs and tissues can be infected [2]. The development of CE cysts is slow and symptoms, if they manifest, are largely non-specific and caused by mass-effect on neighbouring structures or complications (e.g., rupture). A large proportion of CE cases are asymptomatic and have a benign course, but a proportion of cases left undiagnosed develop large cysts and/or complications and require surgical treatment, contrary to early, small cysts which can be successfully treated pharmacologically [3].

CE is a public health problem in areas dedicated to livestock breeding throughout the world. It is estimated that over 1 million people worldwide are infected, with over 800,000 DALYs lost and US$ 3 billion spent yearly for treating human cases and losses to the livestock industry [4]. CE is listed among the Neglected Tropical Diseases (NTDs) targeted for control by the WHO 2021–2030 roadmap on NTDs, aiming for 9 countries having implemented intensified control measures by 2025 and 17 by 2030 [5]. However, control programs for CE are scant [6], and no guidelines exist to guide in practice endemic countries decisions about control needs, baseline prevalence assessment tools, target population(s), practical implementation of one or more control measures, and monitoring and evaluation for decision-making. Only general documents illustrating the possible control measures to be implemented (e.g., dogs’ deworming, safe disposal of livestock offal after slaughter, sheep vaccination, culling of aged sheep, control of free-roaming dogs) are currently available [7,8].

CE prevalence in some areas of Latin America is among the highest in the world [9,10]. In Chile, the average official reported incidence of cases reaching medical attention between 2019–2023 varied between 1.3-3.3 cases per 100,000 inhabitants/year, with the highest figures reached in Aysén (23–56 per 100,000) [11], but these data are largely underestimated due to underreporting, even of hospitalized cases. An effective control program was implemented in the Magallanes region between 1979 and 2004, based on regular deworming of dogs; however, following its discontinuation, the prevalence of infection began to rise again [12]. At present, Chile lacks a structured control program for CE.

**Fig 1:**
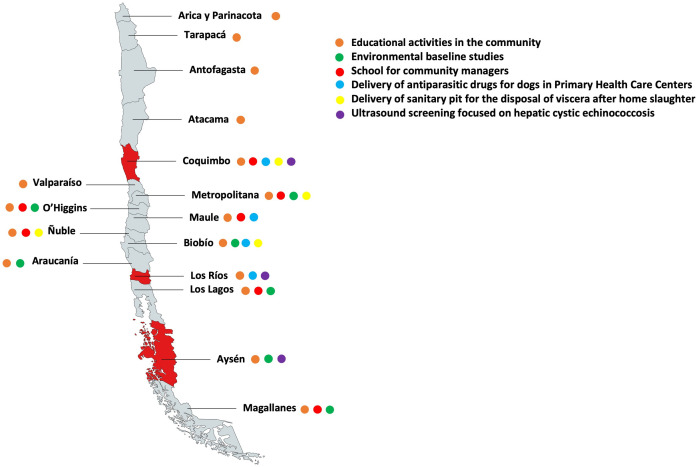
Activities envisaged by the Program for the Control, Monitoring and Prevention of Cystic Echinococcosis developed by the Zoonosis and Vector Control Office of the Ministry of Health of Chile in each region of the country. Ultrasound screening (purple dot) was envisaged in the regions of Coquimbo, Los Ríos and Aysén, where this study was conducted (regions highlighted in red). Figure created from a CC-BY 4.0 license map from mapswire.com (http://mapswire.com/maps/south-america/), modified to incorporate data from the Office for Zoonosis and Vector Control, Division of Public Health Policy and Promotion, Sub-secretariat of Public Health, Ministry of Health, Santiago de Chile, Chile.

In 2004, the Pan American Health Organization (PAHO) launched the Southern Cone Subregional Initiative on Control and Surveillance of Cystic Echinococcosis, aspiring to strengthen and integrate control of CE in the Southern Cone of America into a framework of intercountry cooperation [13]. In 2016, PAHO included CE in the Action Plan for the control of NTDs. More recently, the Regional Program for the Elimination of CE 2020–2029 was launched, aiming to harmonize and monitor progresses of implemented actions in the adhering countries, including Chile [14]. Within this framework, the Action Plan of the Program for the Control, Monitoring and Prevention of CE (later, “Action Plan”) being developed by the Zoonosis and Vector Control Office of the Ministry of Health (MoH) of Chile, includes three components (Prevention, Control, and Monitoring) envisaging various combinations of the following actions, mainly targeted around CE cases (Fig 1): i) health education initiatives; ii) pilot installation of holed plastic barrels for the disposal of livestock viscera after home slaughter, iii) delivery of antiparasitic drugs for dogs in primary health centres of difficult to reach areas, to facilitate procurement by dog owners, and iv) environmental investigation associated to treatment of owned dogs with praziquantel in the household of CE cases notified by hospitals or v) detected by ultrasound (US) screening. This latter US screening activity, currently in the planning phase, is being envisaged in the form of regular abdominal US screening in school-aged children (SAC) in prioritized municipalities of highly endemic regions.

The objective of this study was to implement pilot ultrasound screening activities for the detection of CE in SAC in three municipalities prioritized by the MoH and to estimate the prevalence of CE in this population. The findings aim to inform the MoH about the feasibility of including SAC in the CE screening activities outlined in the Action Plan.

## Methods

### Ethics statement

Ethics approval was granted by the Comité Etico Cientifico de Ciencias de la Salud UC, Pontificia Universidad Católica de Chile, protocol N 230613003 of May 20^th^ 2024. Written consent was sought from the parents or legal representatives of all participants and assent from children. Identifying data of each participant were coupled with the unique participant ID used to identify all study data in a pseudonymized manner. All analyses were carried out identifying the participant by the unique ID code.

### Study design, objectives, and study sites

The study was a cross-sectional study carried out in schools serving the selected municipalities on volunteer school-aged children (6–14 years old), with the main objective of estimating prevalence of andominal CE in this target population in the rural municipalities of Río Hurtado, Coquimbo Region; Paillaco, Los Ríos Region; and Chile Chico, Aysén Region (Fig 1). These locations were selected by the Office for Zoonosis and Vector Control, Division of Public Health Policy and Promotion, Sub-secretariat of Public Health, Ministry of Health, based on results of a K-means algorithm (MoH of Chile, proprietary), based on a series of parameters such as “poverty”, “rurality”, “public water”, “livestock census”, “incidence of cases” and “population of domestic dogs”, ranking them at high risk of CE and therefore prioritized for the implementation of the Action Plan. Data from the MoH of Chile [11] report that in 2023 the number of CE cases and incidence per 100,000 are as follows: Coquimbo N = 2 cases and 1.2/100,000 incidence; Los Rios N = 7 cases and 9.4/100,000 incidence; and Aysen N = 11 cases and 49.5/100.000 incidence.

Personnel from the Regional Secretariat of the Ministry of Health (SeReMi de Salud) of each region coordinated the field activities as part of their institutional mandate.

### Pre-screening educational and capacity-building activities

Before the US screening, written informed consent was sought from the parents or legal tutors of the children, and assent from the children themselves. On the day of the screening, age-targeted educational activities were performed by students of Veterinary Medicine of the Pontificia Universidad Católica de Chile, Santiago, integrated into health education activities on echinococcosis periodically implemented by the local SeReMi de Salud. The educational activities focused on the parasite transmission cycle and measures of infection prevention. In addition, in Valdivia (Los Ríos) and Chile Chico (Aysén), a short training activity for local rural physicians and veterinarians was implemented, encompassing two days of frontal lectures on the biology of the parasite, diagnosis and control in animals and the environment, and diagnosis and treatment in humans. One rural physician working in Río Hurtado, 18 in Los Ríos (who had attended the Diploma in abdominal ultrasound for Primary Care doctors from Universidad de Concepción) and 21 in Aysén (without previous training in US) also joined the US screening sessions to introduce them to the point-of-care use of US for this purpose.

### US screening in school-aged children

In August and September 2024, we carried out a cross-sectional US screening in children of both sexes aged 6–14 years (up to 8^th^ school grade) attending the primary school(s) serving the municipalities, within each region, targeted by the study. Children were eligible if they consented to the exam and their parents or legal guardians consented in writing to their participation. Prior diagnosis of and any treatment for CE were inquired and recorded, but did not constitue an exclusion criterion. On the day of the US exam, children were assigned a unique ID code, which was reported on US files (images and videos) and data files used for data analysis. Only the personnel from the MoH have access to the data file coupling the participant identification data and the ID codes provided to the sonographers and used for the study analyses. Abdominal US was carried out in the municipal primary school(s) during school hours, in a confidential environment, separate from the waiting room where registration and educational activities were carried out. US exams were performed using portable US SonoSite M-Turbo machines (Fujifilm, Seattle, WA, USA) equipped with convex probe; in Los Ríos and Aysén, portable US machines made available by the MoH were also used, and all suspect CE cases were re-assessed and recorded on the SonoSite machines. All participants were examined by sonographers with extensive experience in the diagnosis of CE (FT and TM), who also guided and supervised the US exams performed by rural phisicians as a hands-on training. Teachers and/or parents witnessed the performance of the exam. CE was defined as a focal lesion with stage-specific pathognomonic signs of *E. granulosus* aetiology on US according to the WHO Informal Working Group on Echinococcosis (WHO-IWGE) classification [3]. Briefly, CE1 (unilocular fluid-filled cyst with double-wall), CE2 (fluid-filled cysts with daughter cysts), CE3a (unilocular fluid-filled cysts with detached parasitic layers) and CE3b (daughter cysts in a solid matrix with folded hypoechoic parasitic layers) cysts were classified as active, and CE4 (solid content with folded hypoechoic parasitic layers) and CE5 (CE4 with evident egg-shell calcifications) cysts were classified as inactive. Images and video files of all findings with sure or potential medical relevance were recorded and backed up on two separate storage disks, identified only by the participant ID code.

After US examination, the legal guardian of each participant was provided with a written report about the results of the exam, including any CE or non-CE finding of clear or potential medical importance, and was liaised with the Public Health System for prioritized attention. In case of CE, written recommendation about clinical management, according to the WHO-IWGE [3], was also provided.

### Sample size and data analysis

Based on a defined population of 500 SAC per municipality, and 0.5% prevalence of CE in this age group found in a previous study conducted in the Coquimbo region [15], we calculated that a sample size of 240 SAC per municipality was adequate to estimate a 0.5% prevalence with 0.2% precision and 95% confidence. Results are described as numbers and percentages. Prevalence of human CE at the municipality level is reported with 95% confidence intervals (CI). Results are reported according to the Strengthening the Reporting of Observational Studies in Epidemiology (STROBE) Statement: guidelines for reporting observational studies (S1 STROBE Checklist)

## Results

US screening was carried out on 873 SAC. The details of the cohort are summarized in Table 1 and the source database is freely available at https://zenodo.org/records/15101510.

**Table 1:** Demographic characteristics and US results of the SAC cohort. *For Aysén, the local MoH team established the minimum age for participation at age 7 years, which corresponds to Grade 2. Grade 1 students would have been n = 43. M = males. F = females. CE = cystic echinococcosis.

Municipality (Region)	N SAC per school grade	Sex	CE stages and CE-related US findings	Other US findings
**Río Hurtado (Coquimbo)**	1° N = 33	M = 14 (42%); F = 19 (58%)	–	N = 1 cholelitiasis
	2° N = 30	M = 11 (37%); F = 19 (63%)	–	N = 1 hepatosteatosis
	3° N = 46	M = 20 (43%); F = 26 (57%)	–	–
	4° N = 35	M = 11 (31%); F = 24 (69%)	N = 1 CE3a	N = 4 hepatosteatosis
	5° N = 50	M = 28 (56%); F = 22 (44%)	–	N = 5 hepatosteatosis
	6° N = 41	M = 19 (46%); F = 22 (54%)	–	N = 1 tumoral mass N = 1 hepatosteatosis
	7° N = 51	M = 25 (49%); F = 26 (51%)	–	N = 2 cholelitiasis
	8° N = 48	M = 23 (48%); F25 (52%)	–	N = 2 hepatosteatosis N = 1 cholelitiasis N = 1 hemangioma
	**Total N = 334**	**M = 151 (45%); F = 183 (55%)**	**N = 1 CE (0.3%)**	**N = 13 hepatosteatosis (3.9%)** **N = 4 cholelitiasis(1.2%)** **N = 2 other findings (0.6%)**
**Paillaco (Los Ríos)**	1° N = 33	M = 21 (64%); F = 12 (36%)	–	–
	2° N = 24	M = 16 (67%); F = 8 (33%)	–	N = 2 hepatosteatosis
	3° N = 28	M = 12 (43%); F = 16 (57%)	–	–
	4° N = 28	M = 14 (50%); F = 14 (50%)	–	N = 1 hepatosteatosis
	5° N = 21	M = 13 (62%); F = 8 (38%)	–	N = 2 hepatosteatosis
	6° N = 28	M = 11 (39%); F = 17 (61%)		N = 1 hepatosteatosis N = 1 cholelitiasis
	7° N = 29	M = 17 (59%); F = 12 (41%)	–	N = 3 hepatosteatosis
	8° N = 23	M = 13 (57%); F = 10 (43%)	–	N = 2 hepatosteatosis
	**Total N = 214**	**M = 117 (55%); F = 97 (45%)**	**–**	**N = 11 hepatosteatosis (5.1%)** **N = 1 cholelitiasis (0.5%)**
**Chile Chico (Aysén)**	1° N = 0*	–	–	–
	2° N = 56	M = 25 (45%); F = 31 (55%)	–	N = 1 hepatosteatosis
	3° N = 46	M = 25 (54%); F = 21 (46%)	N = 1 CE3a N = 1 post-surgical cavity	N = 3 hepatosteatosis
	4° N = 51	M = 26 (51%); F = 25 (49%)	–	N = 3 hepatosteatosis
	5° N = 51	M = 24 (47%); F = 27 (53%)	N = 1 CE4 and post-surgical cavity	N = 7 hepatosteatosis
	6° N = 40	M = 21 (53%); F = 19 (47%)	–	–
	7° N = 33	M = 18 (55%); F = 15 (45%)	–	N = 2 hepatosteatosis
	8° N = 48	M = 21 (44%); F = 27 (56%)	–	N = 4 hepatosteatosis N = 3 cholelitiasis
	**Total N = 325**	**M = 160 (49%); F = 165 (51%)**	**N = 2 CE (0.6%)** **N = 1 post-surgical cavity (0.3%)**	**N = 20 hepatosteatosis (6.2%)** **N = 3 cholelitiasis (0.9%)**
**Total N = 873**		**M = 428 (49%); F = 445 (51%)**	**N = 3 CE (0.3%)**	**N = 44 hepatosteatosis (5.0%)** **N = 8 cholelitiasis (0.9%)** **N = 2 other findings (0.2%)**

In Río Hurtado (Coquimbo), 334 children were screened out of total 370 eligible in the target schools (90% attendance) by one experienced sonographer (FT) during four days. In Chile Chico (Aysén) and Paillaco (Los Ríos), 325 childen out of total 400 eligible in the target school (81%) and 214 out of 230 (93%), respectively, were screened during two days in each site by two experienced sconographers (FT and TM).

Three children had hepatic CE cysts (3/873; 0.3%). One case in Río Hurtado, Coquimbo Region (prevalence 0.3%, 95% CI 0.05%-1.7%) who had a 3.3 cm CE3a cyst in the left liver lobe was first diagnosed during this screening. Two known cases were among the SAC screened in Chile Chico, Aysén region (0.6%, 95% CI 0.2%-2.2%), one with a 2 cm CE3a cyst in the IV liver segment and one with a 2.9 cm CE4 cyst in VIII liver segment. The child with the inactive CE4 cysts, as well as another child in the same municipality, had a history of surgery for CE.

The most common other US findings with medical importance were hepatosteatosis (5% overall prevalence, ranging from 3.9%-6.2%) and cholelithiasis (0.9% overall prevalence, ranging from 0.5%-1.2%). One hepatic neoplasm was also first diagnosed in one child.

## Discussion

In this study, we performed pilot US screenings for CE in SAC in three rural municipalities of Chile prioritized by the MoH, to estimate abdominal CE prevalence in this age group and inform the MoH about the possibility of implementing school-based US screening for CE within the activities of the Action Plan of the Program for the Control, Monitoring and Prevention of CE.

Prevalence of CE in SAC ranged from 0.0% in Paillaco (Los Ríos region) to 0.6% (95% CI 0.2-2.2%) in Chile Chico (Aysén region). Due to the high participation of children in each municipality (81–93%), these figures can be considered little prone to self-selection bias deriving from volunteer participation in the screening.

The only CE cyst detected as a first diagnosis in the screened cohort was a small CE3a cyst, which is a transitional stage deriving from the natural evolution from a CE1 cyst (active, early stage) to most likely spontaneous inactivation (CE4 stage) [16,17]. Since small CE3a cysts can be successfully managed using only pharmacological treatment [3], this case illustrates the importance of screening as an early case-finding strategy for CE.

The very low prevalence of CE in SAC found in this study was largely expected from previous data obtained recently in an all-age population screening carried out in the Monte Patria municipality, Coquimbo region [15], adjacent to Río Hurtado, as well as from the resuts of similar studies in other endemic countries [18–21]. Indeed, US-based population screenings consistently show that prevalence of CE is higher in adults than children and that recent infections (arguably reflected by the presence of CE1 cysts) are observed in all age groups, reflecting the cumulative risk of infection to which inhabitants of endemic areas are exposed over time [18–22]. Indeed, despite children are (arguably) believed to be more at risk of infection because of habits such as (unwashed)-hand-to-mouth contact and easy relations with dogs, they are exposed less years to infection risk, in the same area, than an adult, just by anagraphic reasons. Therefore, most likely, the cumulative chances of getting infected through accidental ingestion of eggs are higher in adults than children. In Monte Patria, a valley contiguous to Río Hurtado and with overlapping geographical and socioeconomic features, prevalence in the general population (5–94 years of age) was up to 12% in the rural areas, with a peak prevalence in the 41–60 years age group who also had a large proportion of CE1 stage cysts; the 5–10 years age group had the lowest (<0.5%) prevalence [15]. Thus, low prevalence in SAC should not be misinterpreted as a sign of low transmission and prevalence of CE in the area. Our prevalence results are in line with what officially reported about cases reaching medical attention in Chile, showing that between 2019–2023, CE cases in <15 years of age ranged between 0.6-1.2 per 100,000/year, while adults >60 years of age had the highest incidence (5.3-6.9 per 100,000/year) [11]. In Aysén, in the same years, incidence in <15 years of age ranged between 13.0-49.5 per 100,000/year, the highest among all other regions [11].

Since 2004, Chile is part of the South American Initiative for the Monitoring and Control of Cystic Echinococcosis and has adhered to the PAHO Regional Program for the Elimination of Cystic Echinococcosis 2020–2029 [13,14]. The Action Plan implemented by the Zoonosis and Vector Control Office of the MoH of Chile includes several activities towards CE control. Educational activities, and baseline and post-notification environmental investigations on canine feces to detect *E. granulosus*, are conducted in high-risk areas and following the identification of a human CE case. Praziquantel is provided by the SeReMi team for the deworming of dogs residing in the place, with the instruction to the owners to complete 3 administrations separated by 45 days each. Additionally, perforated plastic barrels (sanitary pits) for the safe disposal of offal from home slaughter are distributed to households, prioritizing those where human or canine infections are detected, as well as in areas with high transmission risk. These measures are variably implemented across Chilean regions (Fig 1). However, the current approach focuses predominantly on areas surrounding reported human CE cases, rather than encompassing region-wide, structured programs aimed at interrupting the parasite’s life cycle comprehensively. Within this framework, the possibility to implement regular US screening in SAC, possibly with the involvement of local non-radiologist rural physicians trained in CE-targeted point-of-care US [23], was pilot tested here.

Screening of SAC by annual abdominal US is currently carried out in the Control Program of Hydatid Disease in the province of Río Negro, Argentina (S1 Table) [23]. This very successful program started in 1980 and currently implements dogs deworming and sheep vaccination as parasite life cycle-interrupting measures. At the start of US application in 1986, screening of children found a prevalence of 5.6%. Based on this baseline figure and on the rationale that infection in children would surely reflect recent infection and thus on-going transmission in the area (no infection can be older that the host, therefore a CE cyst in a child is surely acquired in childhood, while there is some uncertainly about how long before an adult with a CE1 cyst got infected), annual screening of SAC was implemented since then for control program effectiveness evaluation. In general, SAC are often the target of NTD control programs, mainly because the targeted NTDs have higher burden in children, and therefore early diagnosis and treatment is possible; ease of access of school by control program activities also matters (e.g., [24]). Furthermore, specifically in the case of US, incidentalomes requiring medical attention are less frequently encountered in children than in adults, with an expected lower risk of public health care overload.

The MoH of Chile identified SAC < 15 years of age as the target of US screening activities in its Action Plan because of the opportunity to diagnose early CE infections likely requiring only non-invasive treatment, the accessibility of schools, the low risk of public health care overload deriving from the diagnoses made during the US screening in this age group, and the prioritized health attention of children in Chile. The results of our pilot study provided some important insights to this. First, school-based US screening in SAC is technically feasible in the investigated context, adding to the experience of Río Negro [23]. In the target pilot schools, participation was excellent (81–93%), indicating the interest of the communities. Considering that US screening was carried out strictly within school hours, up to 84 SAC per day could be screened (about 16 children/hour) even in sites where local physicians had the chance to flank the experienced physicians and perform hands-on traning on US under their supervision. However, as also evidenced and implemented in Río Negro [23], constant training and monitoring and evaluation of diagnostic performance of local physicians on CE-targeted point-of-care US is essential. Furthermore, constant availability of second opinion or referral to a physician expert in the identification of CE on US are imprescindible, since focal lesions entering in differential diagnosis with CE (in this study, for example, one liver neoplasm and post-surgical cavities) can be present also in children. Second, at least in the specific areas investigated, prevalence of CE in SAC might be too low to allow school-based US screening to be used as a monitoring and evaluation strategy of any envisaged structured control program; baseline data should be gathered in other high risk municipalities to provide a comprehensive epidemiological situation. In this regard, it is to highlight again that, contrary to other NTDs, no actual guidelines exist on how to guide endemic countries in the decision about CE control needs (e.g., cost effectiveness analyses), how to practically implement one or more parasite cycle-interrupting measures, and how to carry out monitoring of control activities (on what host/s species, with what tests). Clearly, such guidelines are urgently needed if WHO and PAHO 2030 targets are to be met. In turn, this would be possible only after the production of at least solid cost-effectiveness data and mathematical modelling of infection transmission and control, and the development of Target Product Profiles for diagnostic assays to be applied on different hosts (dogs, livestock, humans) for both individual diagnosis (in humans) and monitoring and evaluation purposes (mainly dogs and livestock) to direct research in this area. This latter work is currently being carried out by the WHO Diagnostic Technical Advisory Group for Neglected Tropical Diseases, One Health sub-group on neglected zoonotic diseases. More data would also be needed regarding environmental contamination by infective *E. granulosus* eggs; Knowledge, Attitude and Practice studies; and studies investigating site-specific exposure risk habits [25]. Unfortunately, the exclusion of CE from the priority list of any major funding agency dramatically hampers advancements in all these fields and thus towards the achievements of WHO and PAHO 2030 goals.

This study has several limitations, mostly attributable to funding constraints, and logistical limitations due to its embedding in MoH and SeReMi del Salud institutional activities. First, the overall number of SAC screened was small and the investigated target areas were limited, despite in each school the participation rate was excellent. Second, we could not estimate the overall prevalence of CE in the target municipalities, since we could not carry out an all-age population screening, nor we could investigate the overall parasite burden at animal and environmental level. Third, the MoH envisaged activities did not include screening of the household members of children with CE. Fourth, we could not implement a formal cost estimate of the implementation of US screening in schools by rural physicians and of the clinical and economical impact of downstream actions required after diagnoses were made; however, such costs should be available to MoH personnel for further evaluation. In this regard, and considering the low prevalence (hence, low pre-test probability) of CE in SAC, a regular formal assessment of the skills and performance of rural physicians who will carry out US screening of SAC is strongly recommended to limit misdiagnoses to the minimum, which could result in false reassurance (false negatives) or inappropriate actions (false positives). A last limitation, independent from logistics and funding constraint, was that we did not have access to the K-means algorithm of the MoH of Chile, used to rank areas in terms of risk of CE, which is proprietary of the MoH. We strongly hope that this algorithm could be made available to the scientific community, to assess its structure and to evaluate its performance in different geographical areas.

Despite these limitations, this study has the merit to be the second US screening study for CE ever carried out in Chile (after Acosta-Jamett et al. [15], which was focused on a single region) and to provide important insight for the development and implementation of the Chilean Action Plan on CE. Besides, our results showed a relatively high prevalence (5%) of liver steatosis at very young age in the investigated areas. These figures are in line with those reported in two systematic reviews and meta-analysis about pediatric non-alcoholic fatty liver disease (NAFLD), estimating childhood NAFLD and NAFLD in the general population at 7.6% (5.5%-13.3%) and 13% (9%-18%), respectively [26,27]. Also, cholelithiasis prevalence (0.9%) is in line with age-based estimates recently published [28]. Pediatric NAFLD has been associated with prediabetes, hypertension, cardiovascular disease and dyslipidemia [29], and adult NAFLD is a major cause of morbidity and mortality due to cardiovascular, diabetic and neoplastic diseases. A higher risk of gallbladder cancer is also recognized in people with gallbladder stones, with increased risk in South America, and Chile in particular, probably also due to genetic and dietary factors [30]. For this reason, local recommendation on cholelitiasis management impose elective surgery when a diagnosis of cholelithiasis is made, independently of symptoms. Therefore, targeted US screenings could provide the opportunity for early management of both CE and other medically relevant conditions in children, although increased burden on the heath system should be taken into consideration.

In conclusions, we performed pilot US screenings for CE in SAC in three municipalities of Chile prioritized by the MoH, to inform the MoH about the feasibility of targeting this age group within the activities of the Action Plan of the Program for the Control, Monitoring and Prevention of CE. We showed that US screening in SAC is technically feasible. The prevalence of CE in this age group might be too low to allow school-based US screening to be used as a monitoring and evaluation strategy of any envisaged structured control program, but could allow early case-finding and support the implementation of control measures around newly found cases. Especially in the light of the low CE prevalence in SAC, careful training and regular evaluation of skills of rural physicians performing US in this population is strongly recommended, to minimize false positive and negative diagnoses. Furthermore, baseline infection prevalence data should be gathered in other high risk municipalities to provide a comprehensive epidemiological situation. Evidence-based guidelines are urgently need to guide endemic countries in the decision about CE control needs and implementation of control activities, to efficiently advance towards WHO and PAHO 2030 targets for CE.

## Supporting information

S1 STROBE Checklist STROBE Statement—Checklist of items that should be included in reports of cross-sectional studies. (DOCX)

S1 Table Publications concerning the implementation of ultrasound monitoring in control activities for cystic echinococcosis using children as the target population, taken into accunt by the study team before the implementation of this study. (DOCX)
