# Supplementary material for: Ultrasound screening for cystic echinococcosis of school-age children in endemic areas of Chile: A pilot cross-sectional study towards integration in the Regional Program for the Elimination of Cystic Echinococcosis 2020–2029
Source: PLoS Negl Trop Dis. 2025 Jul 21;19(7):e0013301. doi: 10.1371/journal.pntd.0013301 (PMC12303381; doi:10.1371/journal.pntd.0013301)
Supplement: S1 Table — (DOCX) [file pntd.0013301.s002.docx]

**S1 Table**. Publications concerning the implementation of ultrasound monitoring in control activities for cystic echinococcosis using children as the target population, considered before the implementation of this study.

A review of control programs for cystic echinococcsosi (CE) in South America between 1974-2020 was available before the study start. During the study preparation, to expand our background on the implementation of ultrasound (US) monitoring of CE in control activities, using school-aged children (SAC) as the target population, we performed a PubMed search using the keywords “echinococc*”, “hydatid*, “ultraso*”, “monitoring”, “control”, and “children”, with no language limitation, to retrieve papers published until January 2022. The country of implementation, the prevalence of infection in children at the introduction of US in children as a monitoring and evaluation tool, and the rationale at the basis of this target population choice, if available, were obtained for each control program. We retrieved 112 papers, of which 100 were excluded because not dealing with CE (n=19), being clinical studies (n=32), not applying US (n=3), US survey not being in the context of a control programme (n=45), and publication duplicate (n=1). Of the 12 remaining papers, 10 concerned the same control program for CE in Rio Negro (Argentina), one concerned a control program in Tierra del Fuego (Argentinian side) and one was carried out in Turkey but was excluded after analysis of full text since the US survey wasn’t in the context of a control programme. Prevalence of CE on US in SAC were 5.6% in Rio Negro (six years after the start of the control program) and 0.2% in Tierra del Fuego (20 years after the start of the control program). Reported reasons for monitoring infection in children were allowing identification of recent transmissions and diagnosis of infection at early stage.

| **Full reference** | **Control program** |
| --- | --- |
| Arezo M, Mujica G, Uchiumi L, Santillán G, Herrero E, Labanchi JL, Araya D, Salvitti JC, Cabrera M, Grizmado C, Calabro A, Talmon G, Sepulveda L, Galvan JM, Volpe M, Bastin V, Seleiman M, Panomarenko O, Tissot H, Sobrino M, Crowley P, Daffner J, Larrieu E. Identification of potential 'hot spots' of cystic echinococcosis transmission in the province of Río Negro, Argentina. Acta Trop. 2020;204:105341. doi: 10.1016/j.actatropica.2020.105341. | Río Negro, Argentina |
| Larrieu E, Uchiumi L, Salvitti JC, Sobrino M, Panomarenko O, Tissot H, Mercapide CH, Sustercic J, Arezo M, Mujica G, Herrero E, Labanchi JL, Grizmado C, Araya D, Talmon G, Galvan JM, Sepulveda L, Seleiman M, Cornejo T, Echenique H, Del Carpio M. Epidemiology, diagnosis, treatment and follow-up of cystic echinococcosis in asymptomatic carriers. Trans R Soc Trop Med Hyg. 2019;113:74-80. doi: 10.1093/trstmh/try112. Erratum in: Trans R Soc Trop Med Hyg. 2019 May 1;113(5):291. doi: 10.1093/trstmh/try133 | Río Negro, Argentina |
| Salviti JC, Sobrino M, Del Carpio M, Mercapide C, Uchiumi L, Moguilensky J, Moguilansky S, Frider B, Larrieu E. Hidatidosis: Catastro ecográfico en la Provincia de Río Negro 25 anos después del primer catastro [Hydatidosis: Ultrasonographyc screening in the Río Negro Province 25 years after the first screening]. Acta Gastroenterol Latinoam. 2015;45:51-55. | Río Negro, Argentina |
| Larrieu E, Zanini F. Critical analysis of cystic echinococcosis control programs and praziquantel use in South America, 1974-2010. Rev Panam Salud Publica. 2012;31:81-87. doi: 10.1590/s1020-49892012000100012. | Review contro programs for cystic echinococcosis in South America |
| Del Carpio M, Mercapide CH, Salvitti JC, Uchiumi L, Sustercic J, Panomarenko H, Moguilensky J, Herrero E, Talmon G, Volpe M, Araya D, Mujica G, Calabro A, Mancini S, Chiosso C, Labanchi JL, Saad R, Goblirsch S, Brunetti E, Larrieu E. Early diagnosis, treatment and follow-up of cystic echinococcosis in remote rural areas in Patagonia: impact of ultrasound training of non-specialists. PLoS Negl Trop Dis. 2012;6:e1444. doi: 10.1371/journal.pntd.0001444. | Río Negro, Argentina |
| Larrieu E, Del Carpio M, Mercapide CH, Salvitti JC, Sustercic J, Moguilensky J, Panomarenko H, Uchiumi L, Herrero E, Talmon G, Volpe M, Araya D, Mujica G, Mancini S, Labanchi JL, Odriozola M. Programme for ultrasound diagnoses and treatment with albendazole of cystic echinococcosis in asymptomatic carriers: 10 years of follow-up of cases. Acta Trop. 2011;117:1-5. doi: 10.1016/j.actatropica.2010.08.006 | Río Negro, Argentina |
| Zanini F, Suárez C, Pérez H, Elissondo MC. Epidemiological surveillance of cystic echinococcosis in rural population of Tierra del Fuego, Argentina, 1997-2006. Parasitol Int. 2009;58:69-71. doi: 10.1016/j.parint.2008.10.004 | Tierra del Fuego, Argentina |
| Pérez A, Costa MT, Cantoni G, Mancini S, Mercapide C, Herrero E, Volpe M, Araya D, Talmon G, Chiosso C, Vázquez G, Del Carpio M, Santillan G, Larrieu E. Vigilancia epidemiológica de la equinococcosis quística en perros, establecimientos ganaderos y poblaciones humanas en la provincia de Río Negro [Epidemiological surveillance of cystic echinococcosis in dogs, sheep farms and humans in the Rio Negro Province]. Medicina (B Aires). 2006;66:193-200 | Río Negro, Argentina |
| Frider B, Moguilensky J, Salvitti JC, Odriozola M, Cantoni G, Larrieu E. Epidemiological surveillance of human hydatidosis by means of ultrasonography: its contribution to the evaluation of control programs. Acta Trop. 2001;79:219-23. doi: 10.1016/s0001-706x(01)00096-1. | Río Negro, Argentina |
| Del Carpio M, Moguilansky S, Costa M, Panomarenko H, Bianchi G, Bendersky S, Lazcano M, Frider B, Larrieu E. Diagnosis of human hydatidosis. Predictive value of a rural ultrasonographic survey in an apparently healthy population. Medicina (B Aires). 2000;60:466-468 | Río Negro, Argentina |
| Larrieu E, Frider B, Andreani G, Andreani G, Aquino A, De La Fuente R. Hidatidosis humana: ecografía de campo para la determinación de grupos de alto riesgo en la evaluación de un programa de control [Human hydatidosis: field echography for the determination of groups at high risk in the evaluation of a control program]. Rev Inst Med Trop Sao Paulo. 1989;31:267-270. doi: 10.1590/s0036-46651989000400010. | Río Negro, Argentina |
| Frider B, Larrieu E, Vargas F, Odriozzola M, Lester R. Catastro ecográfico, serológico y radiológico en hidatidosis humana. Aporte a un programa de control [An echographic, serologic and radiologic register of human hydatidosis. Contributions to a control program]. Acta Gastroenterol Latinoam. 1985;15:199-211 | Río Negro, Argentina |
